# Treatment of glucocorticoid- induced hyperglycemia in hospitalized patients - a systematic review and meta- analysis

**DOI:** 10.1186/s40842-023-00158-1

**Published:** 2024-01-27

**Authors:** Tristan Struja, Neele Nitritz, Islay Alexander, Kevin Kupferschmid, Jason F. Hafner, Carlos C. Spagnuolo, Philipp Schuetz, Beat Mueller, Claudine A. Blum

**Affiliations:** 1 Department of General Internal and Emergency Medicine, Department of Endocrinology, Diabetology and Metabolism, Medical University Clinic (University of Basel), Kantonsspital Aarau, Tellstrasse 25, Haus 7, Aarau, 5001 Switzerland; 2 Institute for Medical Engineering and Science, Massachusetts Institute of Technology, Cambridge, MA USA; 3 Department of Internal Medicine I, University Hospital of Schleswig-Holstein, Lübeck, Germany; 4 Hormonpraxis Aarau, Praxis für Endokrinologie, Diabetologie und Osteologie, Aarau, Switzerland

**Keywords:** Glucocorticoid-induced Diabetes, Glucocorticoids, Hyperglycemia, Hypoglycemic agents, Insulin

## Abstract

**Purpose:**

Glucocorticoid (GC)-induced hyperglycemia is a frequent issue, however there are no specific guidelines for this diabetes subtype. Although treat-to-target insulin is recommended in general to correct hyperglycemia, it remains unclear which treatment strategy has a positive effect on outcomes. We performed a systematic review and meta-analysis of randomized controlled trials (RCTs) to assess whether treating GC-induced hyperglycemia improves clinical outcomes.

**Methods:**

MEDLINE and EMBASE were systematically searched for RCTs on adults reporting treatment and outcomes of GC-induced hyperglycemia since the beginning of the data bases until October 21, 2023. Glucose-lowering strategies as compared to usual care were investigated.

**Results:**

We found 17 RCTs with 808 patients and included seven trials in the quantitative analysis. Patients with an intensive glucose-lowering strategy had lower standardized mean glucose levels of – 0.29 mmol/l (95%CI -0.64 to -0.05) compared to usual care group patients. There was no increase in hypoglycemic events in the intensively treated groups (RR 0.91, 95%CI 0.70–1.17). Overall, we did not have enough trials reporting clinical outcomes for a quantitative analysis with only one trial reporting mortality.

**Conclusion:**

In GC-induced hyperglycemia, tight glucose control has a moderate effect on mean glucose levels with no apparent harmful effect regarding hypoglycemia. There is insufficient data whether insulin treatment improves clinical outcomes, and data on non-insulin based treatment regimens are currently too sparse to draw any conclusions.

**Systematic review registration:**

Registered as CRD42020147409 at PROSPERO (https://www.crd.york.ac.uk/prospero/) on April 28, 2020

**Supplementary Information:**

The online version contains supplementary material available at 10.1186/s40842-023-00158-1.

## Introduction

Glucocorticoids (GC) are frequently used as anti-inflammatory agents [1], and their use has recently spiked due to the Coronavirus disease 2019 (COVID-19) pandemic [2]. Hyperglycemia is a common side effect of GC treatment and occurs in up to 30% of hospitalized patients, although different incidence rates have been published [3–5]. Guidelines for in-hospital hyperglycemia recommend treat-to-target insulin, but do either not provide specific guidance for GC-induced hyperglycemia or have only weak recommendations due to the missing evidence [6, 7].

After the landmark study of Van den Berghe et al. on critical care patients in 2001 [8], tight in-hospital glucose control was deemed a necessity, until the Normoglycemia in Intensive Care Evaluation–Survival Using Glucose Algorithm Regulation (NICE-SUGAR) study showed that to tight glucose control had a deleterious impact on mortality [9]. The reasons for the increase of mortality are assumed to be attributable to an increase of hypoglycemic events and increased glucose variability [10].

In non- critically ill patients, evidence is scarce. Only one study showed a benefit of short- time glucose control with regard to a reduced infection rate, especially in postoperative patients, however with no influence on mortality [11]. As a consequence, the target glucose values for hospitalized patients not on an intensive care unit has recently been loosened from < 7.8 mmol/l while fasting and < 10 mmol/l postprandially to a more moderate range between 7.8 and 10 mmol/l by the American Diabetes Association [6]. They also make only a general statement on the fact that hyperglycemia may increase mortality and the risk of adverse events such as cardiovascular events and infections.

Reaching the recommended target glucose levels of 7–10 mmol/l in GC-induced hyperglycemia is often difficult to achieve. GC-induced hyperglycemia often has a characteristic pattern of postprandial hyperglycemia, especially around midday, and low nighttime/morning glucose values with consecutively high glucose variability. This bears a high risk of hypoglycemia, along with its concomitant adverse events [12]. The United States (US) Endocrine Society now suggests to use either Neutral Protamin Hagedorn (NPH) insulin or basal-bolus insulin regimens (recommendation 2.1) but this is only a conditional recommendation based on limited evidence [7].

Whether strict treat-to-target treatment of GC-induced hyperglycemia compared to no treatment has a beneficial effect on clinical is not clear, and to our knowledge, it has not been reviewed so far. Available randomized controlled trials (RCTs) and reviews focus mainly on glucose control with insulin [13].

Therefore, we performed a systematic review and meta-analysis of RCTs on the treatment strategies of in-hospital GC-induced hyperglycemia, and its effect on patient outcomes (mortality, cardiovascular events, and infections). Secondary endpoints were treatment effects (glucose variability, mean glucose, time in target range (TIR), and rate of hypoglycemia).

## Materials and methods

### Protocol and eligibility criteria

This review adheres to the Preferred Reporting Items for Systematic Reviews and Meta-Analyses (PRISMA) guidelines. The review protocol was registered in the International prospective register of systematic reviews Prospective Register of Systematic Reviews (PROSPERO) (registration no. CRD42020147409 at https://www.crd.york.ac.uk/prospero/).

We included RCTs which randomized adult in-patients with GC-induced hyperglycemia to two different treatment regimens. We decided to exclude experimental trials with healthy volunteers as this would compare two very distinct and different patient cohorts diluting the conclusion of our analysis. We excluded RCTs on solid organ transplant patients only, as the main immunosuppressive agents have a potentially diabetogenic effect, which differs from the GC effect [14]. Furthermore, studies only on patients with type 1 diabetes were excluded. No restrictions in language, publishing status, or type of literature were made.

GC-induced hyperglycemia was defined as fasting blood glucose greater than 7.0 mmol/l or any blood glucose greater than 11.0 mmol/l occurring after administration of GCs.

### Types of interventions

Trials with interventions consisting of any type of medical treatment of hyperglycemia were included, i.e. different treatment strategies with insulin or addition of an oral antidiabetic agent. We applied no restrictions with respect to the control group treatments, hence they could have either received standard of care treatment or no treatment at all.

### Outcomes

The primary endpoint was mortality and/or any adverse events reported, such as length of hospital stay, readmissions, infections, and the outcome of the underlying disease.

Secondary endpoints were surrogates for glucose control, such as mean or median glucose, TIR, and hypoglycemia.

### Search strategy

We searched the two electronic databases PubMed/Medline and EMBASE from the beginning of each database until October 21, 2023. Search terms included extensive controlled vocabulary and Medical Subject Headings for “GC-induced hyperglycemia” and “treatment” (see Supplementary Appendix for the detailed search strings). We reviewed bibliographies of reviewed articles and searched clinical trials (clinicaltrials.gov) for ongoing or unpublished trials.

### Study selection

Two pairs of reviewers (IA, KK, CCS, JFH) independently screened titles, abstracts, and full texts of any potentially eligible article. The articles were screened first for not meeting the inclusion criteria and second for meeting the exclusion criteria. Discrepancies were resolved through consensus or recourse from a fifth or sixth reviewer (CAB and/or NN).

### Risk of bias assessment of individual studies

All included studies were assessed by two reviewers according to the Scottish Intercollegiate Guidelines Network (SIGN) [15]. The assessment was done by using the checklist accordingly, and the risk of bias was judged to be “high”, “acceptable”, “low”, or “unacceptable”. However, due to the low number of studies, all studies were used for further analysis independent of their risk of bias.

### Data extraction

For studies that fulfilled inclusion criteria, three reviewers independently extracted the data. The final extraction results were checked and discussed among the research team.

From each included paper, we extracted the following information:

- Name, year, country and title of the paper.
- Trial characteristics: design, eligibility criteria, duration, mode of randomization and blinding, intention to treat analysis.
- Number of included patients and patients’ characteristics (type of patients).
- Intervention/ Comparison treatments.
- Outcome measures: primary outcome, secondary outcome, effect size.
- Main results.

Two trial authors were contacted to verify how hypoglycemia was recorded [16, 17]. Radhakutty et al. recorded blood glucose with continuous glucose monitoring on days 1–3, ensuring documentation of all the hypoglycemic events. Beyond day three, there were too few subjects for analysis, as most patients had been discharged from hospital. No data on the incidence of hypoglycemia for the remaining days were available.

Lakhani et al. reported that after a hypoglycemia which was corrected, blood glucose readings were censored for the rest of that day. Furthermore, blood glucose readings from day 1 of randomization were excluded in the final analysis. The authors confirmed this handling, and no additional information on the incidence of hypoglycemia from censored data was given.

### Data synthesis and analysis

Dichotomous data was expressed as risk ratios (RR) with 95% confidence intervals (CI), continuous data as standard mean differences (SMD) with 95% CI. As a test of heterogeneity, the variation in RR and SMD across studies attributable to heterogeneity (I^2^) was computed [18]. As a significant heterogeneity across studies was to be expected, the data was pooled using a random effects model. For each trial, the effect size was plotted by the inverse of its standard error [19]. The symmetry of these ‘‘funnel plots’’ were assessed both visually and formally with Egger’s test to see if the effect decreased with increasing sample size. The statistical analysis was conducted using Stata software v15.1 (Stata Corp., College Station, TX). All significance tests were two-sided, and a p-value of < 0.05 was considered to be statistically significant.

### Sensitivity analysis

We performed an analysis according to post-hoc subgroups based on the abundance of studies. First, subgroups according to the intervention were analyzed. Second, subgroups according to respiratory indication compared to all other indications were analyzed.

## Results

### Systematic search

Our systematic search identified 954 titles and abstracts of potentially eligible studies from the electronic databases (PubMed and Embase) and four additional records. Two of the additional records were gathered from clinicaltrials.gov [20, 21], one record was discovered in the references of another [22], and one was recommended by expert exchange [23]. During the preparation of the manuscript, four newly published RCT on the topic were also included [24–26].

**Fig. 1:**
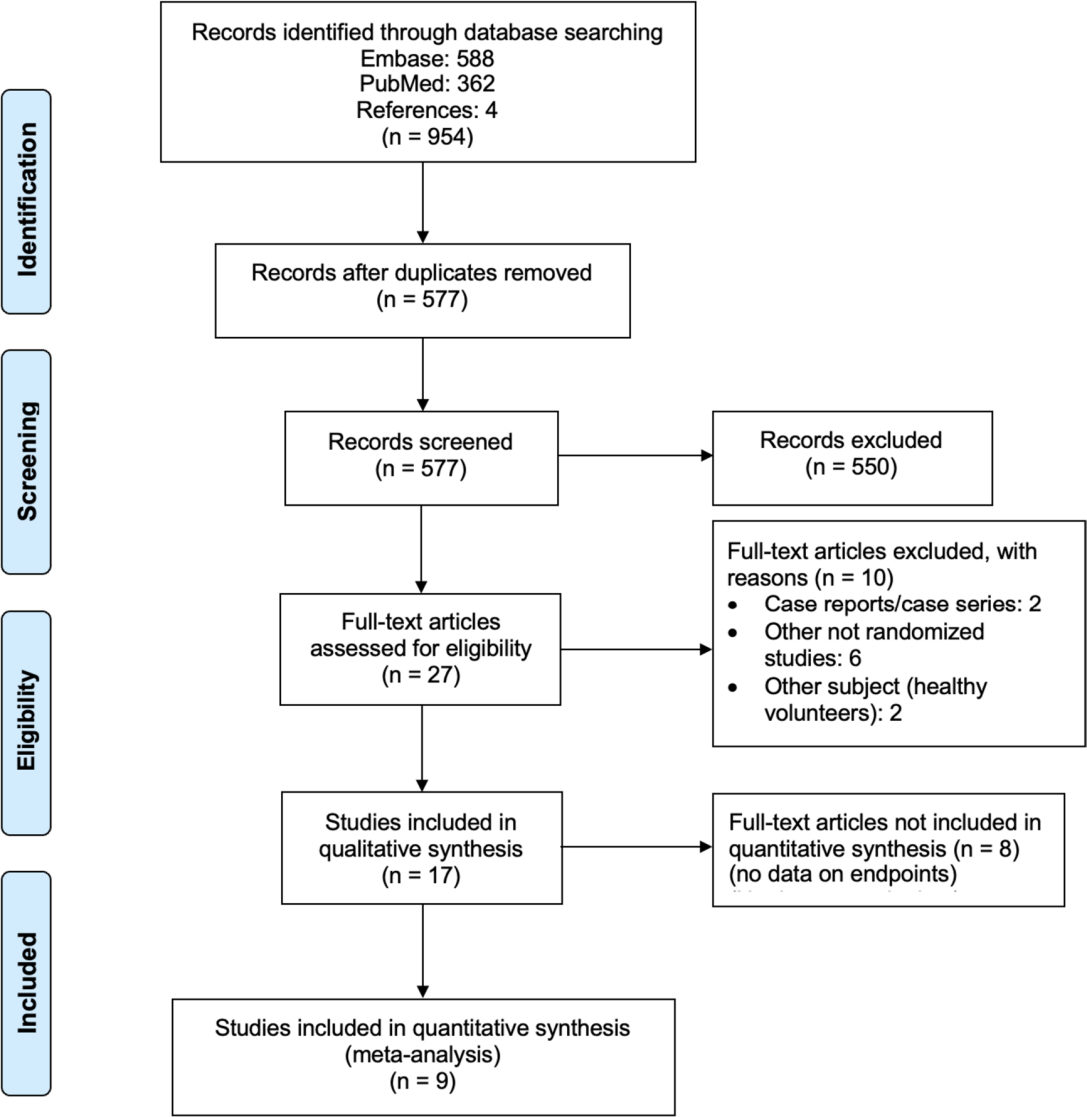
PRISMA flow chart of systematic search. adapted from Moher D, Liberati A, Tetzlaff J, Altman DG, The PRISMA Group (2009). *P*referred *R*eporting *I*tems for *S*ystematic Reviews and *M*eta-*A*nalyses: The PRISMA Statement. PLoS Med 6(7): e1000097. doi:10.1371/journal.pmed1000097

After the removal of duplicates, 577 records were screened. After screening, 550 records were excluded by scanning titles and abstracts, and a further ten records by reviewing the full texts. Seventeen records were eligible for the qualitative analysis. For the quantitative meta-analysis, eight were excluded for having no data on the comparable endpoints. The remaining nine RCTs with a total of 484 patients (range 10 to 103 patients) were included in the final meta-analysis. A flow chart is shown in Fig. 1.

Five of the seventeen included RCTs were multicenter studies. These studies involved a heterogeneous adult population. Four were on patients with hemato-oncologic treatment, four on patients with respiratory diseases, predominantly acute exacerbation of chronic obstructive pulmonary disease (AECOPD), three RCTs were on pregnant women undergoing fetal lung maturation, and three included any GC-induced hyperglycemia in hospitalized patients. Three RCTs were on ambulatory patients receiving GCs for a longer period of time. The studies were published between the years 2000 and 2022. One trial was on patients with a previous diabetes diagnosis, two trials were on patients without diabetes diagnosis only, and all the others were independent of previous diabetes status (with and without previous diabetes). The characteristics of the included RCTs are summarized in Table 1.

**Table 1:** Characteristics of identified randomized controlled trials

Author, year, country	Intervention	Control	Duration of Intervention	Type of Patients	Mean Patient Age	Outcomes reported	Previous Diabetes Status	N
Star J, 2000, USA [27]	High or low-dose insulin	No insulin	3 days	Maternal hyperglycemia during induction of lung maturation	24 years	Glucose (fasting and postprandial)	No diabetes	45
Yamamoto S, 2004, Japan [28]	Pioglitazone	No treatment	6 months	Patients with GC-induced diabetes	59 years	Before and 6 months after pioglitazone oGTT, HbA1c, HOMA-IR	No diabetes	40
Vu K, 2012, USA [22]	Glargine + aspartat	Conventional care	Not reported	Haemato-oncological patients receiving CVAD chemotherapy	52 years	Clinical outcome in acute lymphoblastic leukemia	Any	52
Abdelnour S, 2012, USA [29]	Glargine + lispro + additional lispro	Glargine + lispro + additional NPH	5 days	AECOPD	Not reported	Average daily glucose (day 2–5) and hyperglycemia (10mmol/L), hypoglycemia (< 2.8mmol/L)	Any	19
Ruiz de Adana, 2015, Spain [30]	NPH + bolus	Basal-bolus - insulin	6 days	Respiratory disease	69 years	Mean glucose (premeal and 2 h postprandial), hypoglycemia, glycemic variability, LOS	Type 2 diabetes	53
Gerards MC, 2016, Netherlands [20]	NPH	Sliding scale	3 days	Chemotherapy	67 years	Time in range (3.9–10 mmol/l), proportion of time spent in range, hypoglycemia	Type 2 diabetes	26
Grommesh B, 2016, USA [31]	Add-on NPH	Basal-bolus - insulin	5 days	Not intensive care unit	65 years	Mean glucose, proportion in target range (3.9–10 mmol/l), hypoglycemia, hyperglycemia	Any	61
Hitchings, 2016, U.K. [21]	Metformin	Placebo	1 month	AECOPD	67 years	Mean in-hospital glucose	No diabetes	52
Lakhani OJ, 2017, India [17]	Add-on correction insulin	Basal-bolus - insulin	Not reported, LOS at least 48 h	Any hyperglycemia	54 years	Glucose (fasting and postprandial), hypoglycemic and hyperglycemic events	Any	67
Radhakutty A, 2017, Australia [16]	Isophane + aspartat	Glargine + aspartat insulin	3 days	Hospitalized	72 years	Glycemic control by interstitial continuous glucose monitoring, proportion outside target range (4–10 mmol/l), mean glucose concentration, hypoglycemia, hyperglycemia, HbA1c	No type 1 diabetes	50
Seelig E, 2017, Switzerland [23]	Metformin	Placebo	28 days	Start of ≥ 7.5 mg prednisone-equivalent for ≥ 4 weeks	57 years	Change in area under the curve of oGTT	No diabetes	34
Gerards MC, 2018, Netherlands [32]	Dapagliflozin	Placebo	8 days	AECOPD	72 years	Time in range (3.9–10 mmol/l), LOS	Any	46
Aberer F, 2019, Austria [33]	Basal-bolus - insulin	Sliding scale	41 days	Graft-versus-host disease after allogenic stem cell therapy	57 years	Time in target range (3.9–10 mmol/l)	Not reported	10
Pernicova I, 2020, U.K. [24]	Metformin	Placebo	12 weeks	Inflammatory disease, prednisolone ≥ 20 mg/day for ≥ 4 weeks, ≥ 10 mg/day for the remainder	46 years	Difference in visceral-to-subcutaneous fat area ratio assessed by CT, various changes in metabolic, bone, cardiovascular, and inflammatory parameters	No diabetes	40
Ochola LA, 2020, Kenya [34]	Metformin	Standard of care	4 weeks	Haemato-oncological patients receiving prednisone-based chemotherapy	49.5 years	Hyperglycemia (prediabetes): fasting and 2 h postprandial glucose, metformin effectiveness in lowering glucose	No diabetes	24
Hong JGS, 2021, Malaysia [25]	Metformin	Placebo	3 days	Pregnant women receiving 2 × 12 mg intramuscular dexamethasone 12 h apart	31 years	Hyperglycemic and hypoglycemic episodes, and obstetric outcomes all in mothers,	Any	103
Battarbee AN, 2022, USA [26]	Bolus lispro, or aspart, or regular insulin infusion during labor	No treatment	7 days	Pregnant women 2 × 12 mg intramuscular betamethasone 24 h apart	28 years	Hyperglycemic and hypoglycemic episodes in neonates only, obstetric outcomes in mothers, but no glycemic outcomes	No diabetes	86

*CGM *Interstitial continuous glucose monitoring, *NPH *Neutral Protamin Hagedorn insulin, *AECOPD *Acute exacerbation of chronic obstructive pulmonary disease, *oGTT *Oral glucose tolerance test, *HbA1c *Glycated hemoglobin, *HOMA-IR *Homeostatic model assessment for insulin resistance, *CVAD *Cyclophosphamide, vincristine, doxorubicin, and dexamethasone, *LOS *Length of hospital stay

Interventions consisted mainly of insulin therapies, such as basal - bolus insulin or NPH insulin. Seven RCTs investigated oral antidiabetic drugs (metformin, pioglitazone, and dapagliflozin) as interventions.

Five RCTs investigated intermediate-acting insulin with the hypothesis that the insulin profile matches better with the insulin requirements in GC-induced hyperglycemia.

Patients in the control groups were mostly treated based on usual care, which consisted of sliding scale insulin or basal-bolus insulin. A placebo was used in five of the seven oral antidiabetics trials.

### Qualitative analysis

#### Primary endpoint: mortality and adverse events

Only three trials reported outcome data. Vu et al. reported data on mortality, our primary endpoint [22]. The trial investigated intensive insulin treatment compared to conventional care in patients with acute lymphatic leukemia undergoing a high-dose GC containing chemotherapy regimen. The trial was halted early due to a trend towards a detrimental effect in the intervention group. Even though glucose control was improved in the intervention group compared to the control group, the intervention group showed an excess all-cause 1-year mortality (1-year survival probability 65% in the intervention group and 80% in the control group) and a reduced progression-free survival (intervention 65% vs. control 76%). These outcomes were associated with a higher insulin to C-peptide – ratio, i.e. a higher exogenous insulin application. The authors hypothesize that hyperinsulinemia may have promoted the proliferation of leukemic cells. The intake of thiazolidinediones or metformin was a significant predictor of a lower all-cause mortality and a longer progression-free survival. The authors suggested an improvement of cell metabolism through reduction of hyperinsulinism, insulin resistance, and potentially inhibition of the mammalian target of rapamycin (mTOR) pathway.

Aberer et al. randomized ten patients with acute graft versus host disease after allogeneic hematopoietic stem cell transplantation to either basal-bolus insulin or sliding scale insulin. They reported a median survival time of 105 days (interquartile range (IQR) 39–161) in the intervention group as compared to 136 days (IQR 86–165; p = 0.45) in the control group [33]. Four out of the five patients in each group died within the follow-up of 6 months. In each group, the reasons of death were relapse in one patient, one death due to acute graft-versus-host disease, and two infections. Due to the small number of patients included, underpowering precludes conclusions on whether the intervention had any effect on the outcome.

The third trial to report outcomes was Pernicova et al. [24]. They investigated the use of metformin in patients without diabetes receiving GCs for an inflammatory process at a dose of 20 mg prednisone equivalent for at least four weeks and 10 mg for the consecutive 12 weeks. The main outcome, visceral-to-subcutaneous fat area ratio (assessed by computed tomography scan), was not different at 12 weeks. However, they reported a better metabolic profile in the intervention group. In addition, patients in the intervention group had a markedly lower rate of pneumonia (one, 5% vs. seven, 33%), overall rate of moderate-to-severe infections (two, 10% vs. 11, 52%), and all-cause hospital admissions due to adverse events (one, 5% vs. nine, 43%). As Pernicova et al. did not investigate glucose control itself but the development of metabolic changes due to long-time GC exposure, these outcomes were not put into relation to glucose control in these patients without a history of diabetes. Apparently, metformin is effective for prevention of metabolic complications. The authors discuss that metformin may also additionally modulate the metabolic-immune interplay.

Additionally, three small trials reported only few serious adverse events, which limited their interpretability. Gerards et al. reported 3/12 (8%) events in the intervention group and 2/13 (15%) events in the control group, respectively, in the dapagliflozin trial [32]. In the NPH trial, Gerards et al. reported that six patients experienced an adverse event overall [20]. Hitchings et al. reported 12/36 (33%) vs. 3/18 (17%) serious adverse events not related to the intervention in the metformin vs. control group.

#### Secondary endpoints: glucose control and hypoglycemia

An RCT from 2000 compared pre-emptive high-dose or low-dose insulin to no insulin in pregnant women receiving GCs for fetal lung maturation. Star et al. showed that insulin application at the same time of GC infusion led to a lower rate of hyperglycemia in pregnant women [27], while Hong et al. showed the same using metformin [25]. Battarbee et al. could not show a difference in fetal outcomes with relation to treatment of maternal glucocorticoid-induced hyperglycemia [26].

Aberer, Gerards, and Vu all examined insulin treatment in onco-hematologic patients. They were able to show better glucose control with the more intensive treatment and similarly low rates of hypoglycemia [20, 22, 33]. However, the first two used only sliding scale insulin as the standard of care in the control group, which, as a sole measure, is not the preferred standard of care anymore, due to the lack of efficiency in correcting already high values instead of giving insulin with meals [6, 7].

Ochova et al. showed that adjunct metformin in addition to standard of care in patients receiving glucocorticoids for chemotherapy led to a lower rate of development of prediabetes, defined by 2 h postprandial glucose levels [34].

The trials looking at patients with respiratory symptoms or diseases conducted by Ruiz de Adana et al. and Abdelnour et al. could not demonstrate that their intervention led to a better glucose control [29, 30]. Ruiz de Adana et al. showed that NPH insulin as compared to insulin glargine had a trend towards a lower rate of hypoglycemia. Two trials in AECOPD showed that the use of dapagliflozin and metformin also feasible in these patients [21, 32]. These trials were not designed to show superiority in glucose control.

In the trials recruiting all hospitalized patients experiencing GC-induced hyperglycemia, Radhakutty et al. and Grommesh et al. could not show that intermediate-acting insulin in addition to basal-bolus insulin led to a better glucose control as compared to the classical basal-bolus insulin [16, 31]. Their interventions were safe concerning hypoglycemia, with similar rates between the groups. Mainly, they confirmed the feasibility of intermediate-acting insulin, as its profile of action mimics the pharmacokinetics of prednisone very closely.

The trial by Lakhani et al. was the only insulin intervention study showing significantly better glucose control with an intensive insulin protocol, adapting correction insulin doses to the pharmacology of the received GC, and without an increased rate of hypoglycemia [17].

The RCTs on outpatients investigated the effect of oral antidiabetic drugs in patients receiving long-term GCs.

Yamamoto showed that pioglitazone had a positive effect on hemoglobin A1c (HbA1c) and 2-hour glucose values in oral glucose tolerance testing in patients with previous diabetes receiving GCs [28]. Seelig et al. and Pernicova et al. showed that metformin was effective in preventing metabolic complications in patients without diabetes receiving GCs [23, 24].

### Quantitative analysis: meta-analysis

**Fig. 2:**
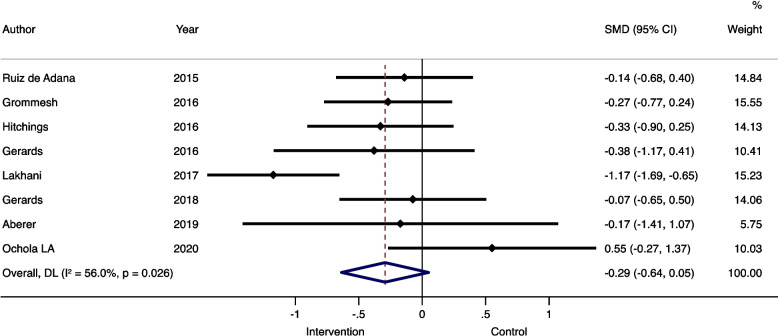
Median/mean blood glucose levels of comparable randomized controlled trials

**Fig. 3:**
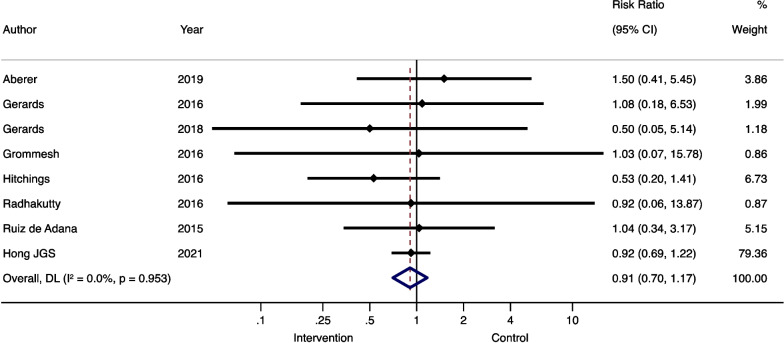
Risk of hypoglycemia of comparable randomized controlled trials

Comparable data was available on mean/median blood glucose and hypoglycemia, our secondary endpoints. Eight studies reported mean/median glucose and nine reported rates of hypoglycemia [17, 20, 21, 25, 30–34]. These studies were included in the quantitative meta-analysis. The meta-analysis showed that the intervention groups had a statistically significant lower overall SMD)in glucose compared to the control groups (SMD – 0.29 95%CI -0.64, -0.05; see also Fig. 2). The rate of hypoglycemia did not differ between the intervention and control groups (RR 0.91, 95%CI 0.70, 1.17; see also Fig. 3).

### Risk of bias assessment

We reviewed risk of bias in each of the thirteen selected trials individually according to the SIGN-Checklist. Of the trials investigating insulin protocols, two used a concealment method for randomization, but the method of blinding was not clear [16, 22]. All trials investigating oral antidiabetic agents reported adequate concealment for randomization and blinding. However, in the trial of Yamamoto et al., only the abstract was available in English, and the full text in Japanese was assessed after translation by an online tool (DeepL.com) and verified by a Japanese-speaking person [28]. Therefore, the trials investigating insulin protocols not reporting an adequate concealment method of randomization or blinding were rated as having an acceptable risk of bias. The adequately blinded trials were rated as having high quality, whereas Yamamoto et al. was rated as having an unacceptable risk of bias, mainly due to uncertainties in translation. Abdelnour was rated of having low quality as only a congress abstract was available [29]. Therefore, there is a potential risk for performance bias. The drop-out rates varied among the trials from 0 to 28% (see also Table 2. Risk of Bias Assessment of Individual Studies).

**Table 2:** Risk of bias assessment of individual studies

Author, year	Appropriate question	Randomi-zation	Adequate Concealment method	Adequate blinding	Similar treatment in groups at start	Only difference is investigated treatment	Standardized, outcome measurement	Drop Outs before study completed	Intention to treat analysis	Overall rating of risk of bias
Star, 2000 [27]	Yes	Yes	No	Can’t say	Yes	Yes	Yes	0%	Yes	Acceptable (+)
Yamamoto, 2004^a^ [28]	No	Can’t say	No	Can’t say	Can’t say	Can’t say	Can’t say	Can’t say	Can’t say	Unacceptable
Vu, 2012 [22]	Yes	Yes	Yes	Can’t say	Yes	Yes	Yes	1.9%	Yes	High quality (++)
Abdelnour, 2012^b^ [29]	Yes	Can’t say	No	Can’t say	Can’t say	Can’t say	Can’t say	Can’t say	Can’t say	Low quality (-)
Ruiz de Adana, 2015 [30]	Yes	Yes	No	No	Yes	Yes	Yes	0%	Yes	Acceptable (+)
Gerards, 2016 [20]	Yes	Yes	Yes	Yes	Can’t say	Can’t say	Yes	15%	Yes	Acceptable (+)
Grommesh, 2016 [31]	Yes	Yes	No	No	Yes	Yes	Yes	15%	Yes	Acceptable (+)
Hitchings, 2016 [21]	Yes	Yes	Yes	Yes	Yes	Yes	Yes	0%	Yes	High quality (++)
Lakhani, 2017 [17]	Yes	Yes	No	No	Yes	Yes	Yes	Control 26% Intervention 28%	Yes	Acceptable (+)
Radhakutty, 2017 [16]	Yes	Yes	Yes	Can’t say	Yes	Yes	Yes	4%	Yes	High quality (++)
Seelig, 2017 [23]	Yes	Yes	Yes	Yes	Yes	Yes	Yes	15%	Yes	High quality (++)
Gerards, 2018 [32]	Yes	Yes	Yes	Yes	Yes	Yes	Yes	0%	Yes	High quality (++)
Aberer, 2019 [33]	Yes	Yes	Can’t say	Can’t say	Yes	Yes	Yes	0%	Yes	Acceptable (+)
Pernicova, 2020 [24]	Yes	Yes	Yes	Yes	Yes	Yes	Yes	Control 22% Intervention 27%	Yes	High quality (++)
Ochola LA, 2020 [34]	Yes	Yes	No	No	Yes	Yes	Yes	Control 15% Intervention 35%	Yes	Acceptable (+)
Hong JGS, 2021 [25]	Yes	Yes	Yes	Yes	Yes	Yes	Yes	Control 27% Intervention 29%	Yes	High quality (++)
Battarbee AN, 2022 [26]	Yes	Yes	Can’t say	No	Yes	Can’t say	Yes	Control 0% Intervention 2%	Yes	Acceptable (+)

^a^Only abstract available in English, full text in Japanese. Translated by online tool (DeepL.com)

^b^Congress abstract only

### Heterogeneity and publication bias

With regards to hypoglycemic event rate, there was a very low heterogeneity among the trials (I^2^ 0%). On the other hand, there was strong evidence for heterogeneity in the glucose lowering effects of the different trials (I^2^ ranging from 45.8 to 67.5%). The funnel plot for hypoglycemia was symmetrical, indicating no hints for small study effects. On the other hand, the funnel plot for SMD glucose displayed a marked asymmetry, pointing to possible effects of small studies and publication bias. This may largely be explained by small study effects and heterogeneity, considering the heterogeneity not only in the indication for GCs, but also in controls and interventions [19] (see also Supplementary Appendix, Figs. 1 and 2).

Formal testing by the Egger’s test was unable to refute the H0 hypothesis of small study effects, albeit this might be due to the small number of studies and the low power of the test.

### Sensitivity analyses

Sensitivity analysis according to indication for GCs (AECOPD vs. all other) implied a smaller effect on glucose control but a larger benefit on the rates of hypoglycemia in AECOPD patients. The effect on glucose control was similar to the overall analysis, whereas there was a trend towards an increased rate of hypoglycemia in non-AECOPD trials. The overall beneficial effect on glucose control was also seen when looking at the different types of intervention, and the risk of hypoglycemia was similar between the intermediate-acting insulin and basal-bolus insulin interventions (see also Supplementary Appendix, Figs. 3, 4, 5, 6).

## Discussion

In this systematic review and meta-analysis on treatment modalities in GC-induced hyperglycemia, we found only scarce data on the effect of treatment on outcomes such as mortality, readmission, or rate of infections. Only one trial in patients with hematologic diseases had all-cause mortality and disease progression as an endpoint [22]. This trial was halted prematurely due to an excess all-cause mortality and lower progression-free survival rate in the intensive insulin group, though the intervention led to better glucose control. Three trials suggested that better glucose control does not necessarily lead to a better outcome while this effect might potentially be limited to patients with malignant diseases. and the interpretation is confounded by the small sample size of these studies [20, 22, 33].

While immediate hyperglycemia bears the risk for acute adverse events such as hyperosmolar coma justifying glucose control, there is virtually no scientific evidence supporting tight glucose control in GC-induced hyperglycemia with regards to long-term outcomes. Both the mode of insulin application as well as the tightness of control for in-hospital hyperglycemia have changed considerably since the early 2000s; from sliding-scale insulin only, to tight glucose control at near-normoglycemic levels, and back to a more moderate glucose control [6, 7, 35, 36]. A standard of care with moderate glucose control and the lowest possible rate of hypoglycemia should be the preferred strategy, as there is only scant evidence in favor of a more rigorous glucose control [11]. A fact that is especially true for GC-induced hyperglycemia as noted in two consensus statements [37, 38].

The second trial investigating patient outcomes was Pernicova et al. [24]. They showed that metformin not only prevented GC-related metabolic and inflammatory side effects in outpatients, but also that this led to a lower rate of infections, especially pneumonia, and consecutively to a lower rate of unplanned hospitalizations. Their results indicate that long-term prevention of metabolic effects by addition of metformin may be beneficial in reducing adverse events [24]. Most guidelines recommend stopping metformin in hospitalized patients. Although metformin is associated with the risk of lactic acidosis, a Cochrane review found no cases of lactic acidosis in 59,321 patient-years of metformin use [39]. In clinically stable patients, there is no good evidence to support routinely stopping metformin at admission, provided that renal glomerular filtration rate is normal [35, 40].

Hitchings et al. showed that the administration of metformin is feasible in hospitalized patients receiving short-term GCs for AECOPD [21]. Their absolute rate of serious adverse events was low, but relative numbers indicated more adverse events in the intervention group (12 of 34 patients, 35%) versus the control group (3 of 18, 16%). On the other hand, patients in the intervention group experienced lower rates of hypoglycemia (18% compared to 33% in the control group). Overall, the small size of the trials does not support the general use of metformin in hospitalized patients receiving GCs.

Our secondary endpoints were glucose control and the rate of hypoglycemia. In summary, the cumulative effect that a more intensive insulin regimen led to better glucose control without excess hypoglycemia, was confirmed. Lakhani et al. showed better glucose control with their intensive insulin treatment protocol. However, this trial is the most controversial [17], as the trial protocol censored all values of a day after hyperglycemia or hypoglycemia, thus introducing a bias towards a more favorable effect for glucose control by potentially under-reporting subsequent events of hypoglycemia and hyperglycemia. The interventions of Gerards, Ruiz de Adana, Radhakutty, and Grommesh tried to antagonize the effect of prednisone on glucose and insulin resistance profiles with intermediate-acting insulin [16, 20, 30, 31], while Lakhani and Abdelnour investigated a protocol aiming at a similar goal with short-acting insulin [17, 29, 41]. Intermediate-acting insulin with a fixed dose or premixed insulin has the advantage of easier application and a better adherence as compared to basal-bolus insulin [42, 43]. The trials on hospitalized patients investigating dapagliflozin and metformin were feasibility trials, whereas the ambulatory metformin and pioglitazone trials showed that long-term metabolic effects were lower in the intervention groups.

Recent trials have challenged the insulin-only strategy in hospitalized patients and have investigated the use of Glucagon-like peptide 1- analogues (GLP-1 analogues) [44–46]. In GC-induced hyperglycemia, data is scarce, however a trial in healthy volunteers [47] and a retrospective observational study [48] show promising results. Not only due to the effect of GLP-1 analogues on insulin resistance and glucose tolerance, but mainly due to the low risk of hypoglycemia.

Another promising alternative to insulin are Sodium Glucose Transporter-2 (SGLT-2) inhibitors, as they have a low risk for hypoglycemia. However as mentioned previously, the experience in GC- induced hyperglycemia is small [32]. One RCT investigating empagliflozin in GC-induced hyperglycemia in in-patients was prematurely ended in 2022 and results are not yet published [49].

A systematic review on the treatment of hyperglycemia of patients with type 2 diabetes receiving systemic glucocorticoid therapy looking at glucose control has yielded similar results: there are few RCTs of limited comparability for treatment of hyperglycemia after GCs [16, 50]. To our knowledge, this is the first systematic review investigating the effect of the treatment of GC-induced hyperglycemia on mortality and adverse events.

### Limitations and strengths

The primary limitation of this systematic review lies in the paucity of accessible evidence, particularly the scarcity of high-quality RCTs on this specific topic. Additionally, the interpretation of existing studies is complicated by significant heterogeneity in terms of geographical location, study settings, and variations in measurement and treatment protocols. While a meta-analysis facilitates the amalgamation of available evidence, making it more readily accessible, it cannot replace the insights obtained from meticulously conducted clinical trials. Moreover, the absence of patient-level data and the omission of network analysis are noteworthy limitations. A particular strength of our work is that this review is the first comprehensive investigation into patient outcomes, including aspects such as mortality and adverse events.

## Conclusion

There is insufficient data whether insulin treatment has an effect on patient-specific outcomes (i.e. mortality, and other adverse events) in GC-induced hyperglycemia.

Intermediate-acting insulin may be equally effective to basal-bolus insulin in reducing GC-induced hyperglycemia and preventing hypoglycemic events. Data on non-insulin-based treatment regimens are currently too sparse to draw any conclusions.

### Supplementary Information

**Additional file 1: Supplementary Appendix.** **Supplemental Figure 1**. Funnel plot for risk of hypoglycemia **Supplemental Figure 2**. Funnel plot for glucose values. **Supplemental Figure 3.** Splitted Forest plots, glucose in AECOPD-studies vs. all other. **Supplemental Figure 4.** Splitted Forest plots, hypoglycemia in AECOPD-studies vs. all other. **Supplemental Figure 5.** Splitted Forest plots, glucose according to type of intervention. **Supplemental Figure 6.** Splitted Forest plots, hypoglycemia according to type of intervention

## Data Availability

We only used publicly available sources. Interested researches can contact the corresponding author to obtain our data.

## Abbreviations

AECOPD: Acute exacerbation of chronic obstructive pulmonary disease
CI: Confidence Interval
GC: Glucocorticoid
GLP-1: Glucagon-like peptide 1
IQR: Interquartile range
NPH: Neutral Protamin Hagedorn insulin
RCT: Randomized controlled trial
RR: Risk Ratio
TIR: Time in target range
SIGN: Scottish Intercollegiate Guidelines Network
SGLT-2: Sodium Glucose Transporter 2
SMD: Standard mean difference
