# Supplementary material for: Treatment of glucocorticoid- induced hyperglycemia in hospitalized patients - a systematic review and meta- analysis
Source: Clin Diabetes Endocrinol. 2024 Jan 27;10:8. doi: 10.1186/s40842-023-00158-1 (PMC10821212; doi:10.1186/s40842-023-00158-1)
Supplement: Supplementary file 1 — Additional file 1: Supplementary Appendix. Supplemental Figure 1. Funnel plot for risk of hypoglycemia Supplemental Figure 2. Funnel plot for glucose values. Supplemental Figure 3. Splitted Forest plots, glucose in AECOPD-studies vs. all other. Supplemental Figure 4. Splitted Forest plots, hypoglycemia in AECOPD-studies vs. all other. Supplemental Figure 5. Splitted Forest plots, glucose according to type of intervention. Supplemental Figure 6. Splitted Forest plots, hypoglycemia according to type of intervention [file 40842_2023_158_MOESM1_ESM.docx]

**Supplementary Appendix**

**Supplemental Figure 1. Funnel plot for risk of hypoglycemia**

**Supplemental Figure 2. Funnel plot for glucose values**

**Supplemental Figure 3. Splitted Forest plots, glucose in AECOPD-studies vs. all other**

**Supplemental Figure 4. Splitted Forest plots, hypoglycemia in AECOPD-studies vs. all other**

**Supplemental Figure 5. Splitted Forest plots, glucose according to type of intervention**

**Supplemental Figure 6. Splitted Forest plots, hypoglycemia according to type of intervention**

**Complete Search Strings**

**PubMed**

Search (((((Hypoglycemic Agent*[Title/Abstract] OR Hypoglycemic drug*[Title/Abstract] OR Hyperglycemia therap*[Title/Abstract] OR hyperglycemia treatment*[Title/Abstract] OR Insulin[Title/Abstract] OR Insulin Glargin[Title/Abstract] OR Dipeptidyl-Peptidase IV Inhibitor*[Title/Abstract] OR Metformin*[Title/Abstract] OR Thiazolidinedion*[Title/Abstract]))) OR ((((((((((( "Hyperglycemia/drug therapy"[Mesh] OR "Hyperglycemia/therapy"[Mesh] ))) OR (( "Insulin/administration and dosage"[Mesh] OR "Insulin/therapeutic use"[Mesh] ))) OR (( "Insulin Glargine/administration and dosage"[Mesh] OR "Insulin Glargine/therapeutic use"[Mesh] ))) OR (( "Hypoglycemic Agents/administration and dosage"[Mesh] OR "Hypoglycemic Agents/therapeutic use"[Mesh] ))) OR (( "Dipeptidyl-Peptidase IV Inhibitors/administration and dosage"[Mesh] OR "Dipeptidyl-Peptidase IV Inhibitors/therapeutic use"[Mesh] ))) OR (( "Metformin/administration and dosage"[Mesh] OR "Metformin/therapeutic use"[Mesh] ))) OR (( "Thiazolidinediones/administration and dosage"[Mesh] OR "Thiazolidinediones/therapeutic use"[Mesh] )))) AND Humans[Mesh]))) AND ((((((((((("Hyperglycemia/chemically induced"[Mesh]) OR "Diabetes Mellitus/chemically induced"[Mesh:NoExp]) OR "Diabetes Mellitus, Type 2/chemically induced"[Mesh:NoExp])) AND Humans[Mesh])) AND (((((((((((("Immunosuppressive Agents/adverse effects"[Mesh:NoExp]) OR "Anti-Inflammatory Agents/adverse effects"[Mesh:NoExp]) OR "Glucocorticoids/adverse effects"[Mesh]) OR "Betamethasone/adverse effects"[Mesh]) OR "Dexamethasone/adverse effects"[Mesh]) OR "Methylprednisolone/adverse effects"[Mesh]) OR "Prednisolone/adverse effects"[Mesh]) OR "Prednisone/adverse effects"[Mesh]) OR "Hydrocortisone/adverse effects"[Mesh]) OR "Triamcinolone/adverse effects"[Mesh])) AND Humans[Mesh]))))) OR (((((((Immunosuppressive Agents*[Title/Abstract] OR Anti-Inflammatory Agent*[Title/Abstract] OR Steroid[Title/Abstract] OR glucocorticoid*[Title/Abstract] OR corticosteroid*[Title/Abstract] OR Betamethason*[Title/Abstract] OR Dexamethason*[Title/Abstract] OR Methylprednisolon*[Title/Abstract] OR Prednisolon*[Title/Abstract] OR Prednison*[Title/Abstract] OR Hydrocortison*[Title/Abstract] OR Triamcinolon*[Title/Abstract])) AND ((diabetes mellitus[Title/Abstract] OR diabetes[Title/Abstract] OR Hyperglycem*[Title/Abstract] OR hyperglycaem*[Title/Abstract]))) AND ((adverse effect[Title/Abstract] OR adverse effects[Title/Abstract])))) OR ((((steroid related[Title/Abstract] OR corticosteroid related[Title/Abstract] OR glucocorticoid related[Title/Abstract]))) AND ((diabetes mellitus[Title/Abstract] OR diabetes[Title/Abstract] OR Hyperglycem*[Title/Abstract] OR hyperglycaem*[Title/Abstract])))) OR ((Steroid Diabet*[Title/Abstract] OR GIDM[Title/Abstract] OR GC-DM[Title/Abstract] OR (GID[Title/Abstract] AND diabetes[Title/Abstract]) OR "glucocorticoid induced diabetes"[Title/Abstract] OR "glucocorticoid induced diabetes mellitus"[Title/Abstract] OR "glucocorticoid induced hyperglycemia"[Title/Abstract] OR "corticosteroid induced diabetes"[Title/Abstract] OR "corticosteroid induced diabetes mellitus"[Title/Abstract] OR "corticosteroid induced hyperglycemia"[Title/Abstract] OR "steroid induced diabetes"[Title/Abstract] OR "steroid induced diabetes mellitus"[Title/Abstract] OR "steroid induced hyperglycemia"[Title/Abstract] OR "steroid induced hyperglycaemia"[Title/Abstract]))))

**Embase**

(('steroid diabet*':ab,ti OR 'gidm':ab,ti OR 'gc-dm':ab,ti OR '(gid':ab,ti) AND 'diabetes)':ab,ti OR 'glucocorticoid induced diabetes':ab,ti OR 'glucocorticoid induced diabetes mellitus':ab,ti OR 'glucocorticoid induced hyperglycemia':ab,ti OR 'corticosteroid induced diabetes':ab,ti OR 'corticosteroid induced diabetes mellitus':ab,ti OR 'corticosteroid induced hyperglycemia':ab,ti OR 'steroid induced diabetes':ab,ti OR 'steroid induced diabetes mellitus':ab,ti OR 'steroid induced hyperglycemia':ab,ti OR 'steroid induced hyperglycaemia':ab,ti OR (('(steroid related':ab,ti OR 'corticosteroid related':ab,ti OR 'glucocorticoid related)':ab,ti) AND ('(diabetes mellitus':ab,ti OR 'diabetes':ab,ti OR 'hyperglycem*':ab,ti OR 'hyperglycaem*)':ab,ti)) OR (('(immunosuppressive agents*':ab,ti OR 'anti-inflammatory agent*':ab,ti OR 'steroid':ab,ti OR 'glucocorticoid*':ab,ti OR 'corticosteroid*':ab,ti OR 'betamethason*':ab,ti OR 'dexamethason*':ab,ti OR 'methylprednisolon*':ab,ti OR 'prednisolon*':ab,ti OR 'prednison*':ab,ti OR 'hydrocortison*':ab,ti OR 'triamcinolon*)':ab,ti) AND ('(adverse effect':ab,ti OR 'adverse effects)':ab,ti) AND ('(diabetes mellitus':ab,ti OR 'diabetes':ab,ti OR 'hyperglycem*':ab,ti OR 'hyperglycaem*)':ab,ti)) OR (('corticosteroid'/exp/dd_ae OR 'immunosuppressive agent'/exp/dd_ae OR 'immunosuppressive agent'/dd_ae OR 'antiinflammatory agent'/exp/dd_ae OR 'antiinflammatory agent'/dd_ae OR 'dexamethasone'/exp/dd_ae OR 'methylprednisolone'/exp/dd_ae OR 'prednisolone'/exp/dd_ae OR 'prednisone'/exp/dd_ae OR 'hydrocortisone'/exp/dd_ae OR 'triamcinolone'/exp/dd_ae) AND 'chemically induced'/exp AND ('hyperglycemia'/exp OR 'diabetes mellitus'/exp OR 'diabetes mellitus'/de OR 'non insulin dependent diabetes mellitus'/exp OR 'non insulin dependent diabetes mellitus'/de) AND [humans]/lim)) AND (('corticosteroid'/exp/dd_ae OR 'immunosuppressive agent'/exp/dd_ae OR 'immunosuppressive agent'/dd_ae OR 'antiinflammatory agent'/exp/dd_ae OR 'antiinflammatory agent'/dd_ae OR 'dexamethasone'/exp/dd_ae OR 'methylprednisolone'/exp/dd_ae OR 'prednisolone'/exp/dd_ae OR 'prednisone'/exp/dd_ae OR 'hydrocortisone'/exp/dd_ae OR 'triamcinolone'/exp/dd_ae) AND 'chemically induced'/exp AND ('hyperglycemia'/exp OR 'diabetes mellitus'/exp OR 'diabetes mellitus'/de OR 'non insulin dependent diabetes mellitus'/exp OR 'non insulin dependent diabetes mellitus'/de) AND [humans]/lim OR (('steroid diabet*':ab,ti OR 'gidm':ab,ti OR 'gc-dm':ab,ti OR '(gid':ab,ti) AND 'diabetes)':ab,ti) OR 'glucocorticoid induced diabetes':ab,ti OR 'glucocorticoid induced diabetes mellitus':ab,ti OR 'glucocorticoid induced hyperglycemia':ab,ti OR 'corticosteroid induced diabetes':ab,ti OR 'corticosteroid induced diabetes mellitus':ab,ti OR 'corticosteroid induced hyperglycemia':ab,ti OR 'steroid induced diabetes':ab,ti OR 'steroid induced diabetes mellitus':ab,ti OR 'steroid induced hyperglycemia':ab,ti OR 'steroid induced hyperglycaemia':ab,ti OR (('(steroid related':ab,ti OR 'corticosteroid related':ab,ti OR 'glucocorticoid related)':ab,ti) AND ('(diabetes mellitus':ab,ti OR 'diabetes':ab,ti OR 'hyperglycem*':ab,ti OR 'hyperglycaem*)':ab,ti)) OR (('(immunosuppressive agents*':ab,ti OR 'anti-inflammatory agent*':ab,ti OR 'steroid':ab,ti OR 'glucocorticoid*':ab,ti OR 'corticosteroid*':ab,ti OR 'betamethason*':ab,ti OR 'dexamethason*':ab,ti OR 'methylprednisolon*':ab,ti OR 'prednisolon*':ab,ti OR 'prednison*':ab,ti OR 'hydrocortison*':ab,ti OR 'triamcinolon*)':ab,ti) AND ('(adverse effect':ab,ti OR 'adverse effects)':ab,ti) AND ('(diabetes mellitus':ab,ti OR 'diabetes':ab,ti OR 'hyperglycem*':ab,ti OR 'hyperglycaem*)':ab,ti))) AND ('hypoglycemic agent*':ab,ti OR 'hypoglycemic drug*':ab,ti OR 'hyperglycemia therap*':ab,ti OR 'hyperglycemia treatment*':ab,ti OR 'insulin':ab,ti OR 'insulin glargin':ab,ti OR 'dipeptidyl-peptidase iv inhibitor*':ab,ti OR 'metformin*':ab,ti OR 'thiazolidinedion*':ab,ti OR 'sodium glucose cotransporter 2 inhibitor*':ab,ti OR 'sglt 2 inhibitor*':ab,ti OR 'glp 1 ra':ab,ti OR 'glucagon like peptide 1 receptor antagonist':ab,ti OR 'glucagon like peptide 1 receptor agonist':ab,ti OR 'glp 1 r agonist':ab,ti OR 'glp 1 r antagonist':ab,ti OR 'sulfonylurea*':ab,ti OR (('hyperglycemia'/exp/dm_dt,dm_th OR 'insulin'/exp/dd_ad,dd_cr,dd_dt OR 'insulin glargine'/exp/dd_ad,dd_cr,dd_dt OR 'antidiabetic agent'/exp/dd_ad,dd_cr,dd_dt OR 'dipeptidyl peptidase iv inhibitor'/exp/dd_ad,dd_cr,dd_dt OR 'metformin'/exp/dd_ad,dd_cr,dd_dt OR '2,4 thiazolidinedione derivative'/exp/dd_ad,dd_cr,dd_dt OR 'sodium glucose cotransporter 2 inhibitor'/exp/dd_ad,dd_cr,dd_dt OR 'glucagon like peptide 1 receptor agonist'/exp/dd_ad,dd_cr,dd_dt OR 'sulfonylurea'/exp/dd_ad,dd_cr,dd_dt) AND [humans]/lim AND 'human'/de))
